# Editorial: Food literacy and healthy diets in childhood and adolescence

**DOI:** 10.3389/fnut.2024.1366912

**Published:** 2024-01-25

**Authors:** Maha Hoteit, Radwan Qasrawi, Reema Tayyem

**Affiliations:** ^1^Food Sciences Unit, National Council for Scientific Research-Lebanon (CNRS-L), Beirut, Lebanon; ^2^Department of Computer Science, Al-Quds University, Jerusalem, Palestine; ^3^Department of Computer Engineering, Istinye University, Istanbul, Türkiye; ^4^Department of Human Nutrition, College of Health Sciences, Qatar University, Doha, Qatar

**Keywords:** food literacy, nutrition literacy, education, children, adolescents

The global shift in food systems has made processed food, which is nutrient-poor and energy-dense, readily available and convenient for individuals across all age groups, with a particular focus on children. This phenomenon significantly influences food choices, leading to the disruption of dietary patterns. Two crucial concepts related to health literacy play a pivotal role in supporting the promotion of healthy dietary habits: nutrition literacy (NL) and food literacy (FL). The definition of NL is “*the degree to which individuals have the capacity to obtain, process, and understand nutrition information and skills needed in order to make appropriate nutrition decisions*” (1); where FL has been defined as “*the scaffolding that empowers individuals, households, communities or nations to protect diet quality through change and strengthen dietary resilience over time. It is composed of a collection of inter-related knowledge, skills and behaviors required to plan, manage, select, prepare and eat food to meet needs and determine intake*” (2).

The support for developing NL and FL among children begins in their preschool years, shaping their food choices and building food-related skills and practices that will support their habits later in life. The objective of this research is to compile papers that enhance our understanding of the impact of implementing and developing NL and FL among infants, children, and school-age students on their health-related patterns. The Research Topic consists of 11 articles.

A review paper by Hoteit, Mansour et al. reveals the prevalence of NL and FL in 10 Arab countries. Approximately 28% of Arab adolescents exhibited poor NL, with 60% of their parents being food illiterate. Variations were observed in different countries, such as Qatar (44%), Lebanon (37.4%), and Saudi Arabia (34.9%). Several factors, including adolescents' age, gender, education level, primary caregivers, employment status, and the inclusion of nutrition education in schools, predicted the nutrition literacy levels of Arab adolescents. Additionally, parental weight status, health status, parental food literacy level, and the number of children per household were significant determinants. Hoteit, Mohsen et al.'s data indicate that FL and NL can mitigate malnutrition in Lebanon. In Saudi Arabia, Bookari's (a) study demonstrated that approximately 46% of parents of adolescents had poor food literacy, influencing their health status. Parental education level and socioeconomic status were also associated with food illiteracy.

Another study by Bookari (b) in Saudi Arabia revealed that nearly half of the adolescents (44.6%) had poor nutrition literacy. Factors such as gender, residential area, weight status, school status, and caregivers other than parents influenced FL and NL. Culinary lessons focusing on vegetables in schools were shown by Policastro et al. to drive dietary behavior change and self-efficacy to cook, impacting the acceptance of vegetables. Cao et al.'s study in primary and secondary schools indicated that children (8–15 years old) with gender identity concerns exhibited higher frequencies of unhealthy behaviors, particularly in consuming carbonated beverages. Ding et al. emphasized the importance of providing dairy products to infants and young children, which led to improved intake of essential nutrients.

In Egypt, Bonsu and Addo reported that one in every six children under 5 years was overweight or obese. Determinants included high birth weights, consumption of large portions of protein foods, and mothers in the rich wealth quintile. Lack of education on FL and breastfeeding negatively affected the health status of these children, as shown in Alotiby's systematic review linking breastfeeding to protection against chronic diseases like obesity and diabetes mellitus.

Tang et al. highlighted the association between the lack of FL among mothers in China and the prevalence of feeding difficulties in young children. The gap in FL and NL affected feeding styles, contributing to issues such as forcing children to eat and allowing play during mealtime. Finally, Chuang et al. investigated the dietary profile of pediatric obstructive sleep apnea (OSA) patients, emphasizing the importance of integrating a FL system for parents to reduce unhealthy patterns among children, a significant factor in OSA.

In conclusion, this book provides readers with updated data and studies from multiple countries, presenting a wealth of new information on FL and NL. The papers published in this e-book underscore that there are still many aspects within the realm of FL and NL that require further clarification and understanding.

## Author contributions

MH: Conceptualization, Data curation, Formal analysis, Funding acquisition, Investigation, Methodology, Project administration, Resources, Software, Supervision, Validation, Visualization, Writing – original draft, Writing – review & editing. RQ: Conceptualization, Data curation, Formal analysis, Funding acquisition, Investigation, Methodology, Project administration, Resources, Software, Supervision, Validation, Visualization, Writing – original draft, Writing – review & editing. RT: Conceptualization, Data curation, Formal analysis, Funding acquisition, Investigation, Methodology, Project administration, Resources, Software, Supervision, Validation, Visualization, Writing – original draft, Writing – review & editing.

## References

[1] Silk K Sherry J Betal W. Increasing nutrition literacy: testing the effectiveness of print, website, and game modalities. J Nutr Educ Behav. (2008) 40:3–106. 10.1016/j.jneb.2007.08.012 18174098

[2] Vidgen HA Gallegos D. Defining food literacy and its components. Appetite. (2014) 76:50–9. 10.1016/j.appet.2014.01.010 24462490

